# Automated classification of dense calcium tissues in gray-scale intravascular ultrasound images using a deep belief network

**DOI:** 10.1186/s12880-019-0403-8

**Published:** 2019-12-30

**Authors:** Juhwan Lee, Yoo Na Hwang, Ga Young Kim, Ji Yean Kwon, Sung Min Kim

**Affiliations:** 1 Department of Biomedical Engineering, Case Western Reserve University, 10900, Euclid Avenue, Cleveland, OH 44106 USA; 2 Department of Medical Biotechnology, Dongguk University-Bio Medi Campus, (10326) 32, Dongguk-ro, Ilsandong-gu, Goyang-si, Gyeonggi-do Republic of Korea; 3 Department of Medical Devices Industry, Dongguk University-Seoul, (04620) 30, Pildong-ro 1-gil, Jung-gu, Seoul, Republic of Korea

**Keywords:** Intravascular ultrasound, Dense calcium, Textural feature, Deep belief network

## Abstract

**Background:**

IVUS is widely used to quantitatively assess coronary artery disease. The purpose of this study was to automatically characterize dense calcium (DC) tissue in the gray scale intravascular ultrasound (IVUS) images using the image textural features.

**Methods:**

A total of 316 Gy-scale IVUS and corresponding virtual histology images from 26 patients with acute coronary syndrome who underwent IVUS along with X-ray angiography between October 2009 to September 2014 were retrospectively acquired and analyzed. One expert performed all procedures and assessed their IVUS scans. After image acquisition, the DC candidate and corresponding acoustic shadow regions were automatically determined. Then, nine image-base feature groups were extracted from the DC candidates. In order to reduce the dimensionalities, principal component analysis (PCA) was performed, and selected feature sets were utilized as an input for a deep belief network. Classification results were validated using 10-fold cross validation.

**Results:**

The dimensionality of the feature map was efficiently reduced by 50% (from 66 to 33) without any performance decrease using PCA method. Sensitivity, specificity, and accuracy of the proposed method were 92.8 ± 0.1%, 85.1 ± 0.1%, and 88.4 ± 0.1%, respectively (*p* < 0.05). We found that the window size could largely influence the characterization results, and selected the 5 × 5 size as the best condition. We also validated the performance superiority of the proposed method with traditional classification methods.

**Conclusions:**

These experimental results suggest that the proposed method has significant clinical applicability for IVUS-based cardiovascular diagnosis.

## Background

Atherosclerotic cardiovascular diseases are known to be the leading cause of morbidity and mortality in developed countries. Atherosclerotic plaques are typically composed of lipids, inflammatory cells, and calcium deposits [1] and the disruption of these plaques depends on the composition of the tissue components, with tissue compositions being classifiable calcified, fibrous, fibro-lipid, and necrotic [2]. Therefore, analyzing plaque composition is an important procedure that will allow the physicians to acquire an overall status of the disease and determine the appropriate interventional therapies.

Intravascular ultrasound (IVUS) is a catheter-based imaging modality that provides real-time tomographic views of the coronary arteries and allows for a detailed visualization of the plaque [1]. The use of IVUS has been crucial for the quantitative assessment of coronary artery disease. Provided a gray-scale IVUS image, expert physicians are able to manually determine the vessel borders from lumen to media-adventitia, where the atherosclerotic plaques are distributed, and then differentiate between the plaque components using their echogenicity. The primary limitation of the gray-scale IVUS in the plaque characterization is that the gray-level appearance (echogenicity) does not correspond well with the plaque constituents [3]. For example, it is very difficult to differentiate between fibrous and fibro-lipid tissues only using the observable characteristics owing to similar intensity ranges. Therefore, the manual identification of the cross-sectional images is not straightforward and is susceptible to inter-observer and intra-observer reliability.

Virtual histology (VH) is a fully automated and clinically available technique that characterizes plaque components by exploiting the reflected ultrasound radio-frequency (RF) signals. VH-IVUS is able to identify the four plaque types including fibrous tissue (FT), fibro-fatty tissue (FFT), necrotic core (NC), and dense calcium (DC), using a color-coded map [4, 5]. Among these tissues, the amount of DC is an important indicator of atherosclerotic disease [6], as it is strongly associated with the overall risk of acute myocardial infarction as well as with complications and success rates following percutaneous coronary intervention [7, 8]. The calcified plaque also enables inference of the entire plaque burden and overall disease status [9]. Therefore, the quantitative analysis of the DC can be used to reduce the risk of occlusions and operations.

In order to precisely and efficiently detect the calcified region, several automated plaque characterization approaches have been suggested in terms of the RF signal, image texture, and combined feature groups. The biggest benefits of the RF-based approaches are the reduction of the feature dimensionality and the largest increase in computational speed [5, 10–20]. Moreover, as previously shown in various histologic analyses, these methods revealed outstanding classification results for vessel compositions. However, all of the RF-based methods suffer from the reduced longitudinal resolution caused by its electrocardiogram (ECG)-gated acquisition [1, 21]. Therefore, it is not allowed to analyze all image slices and construct a three-dimensional map for the whole vessel region. On the other hand, the image texture-based approaches are able to analyze all the gray-scale image frames [1, 2, 21–32]. Textural analysis approaches attempt to quantify the perceived texture of lesion mostly based on the spatial characteristics or echogenicity. These methods can be implemented into any types of IVUS images which are obtained from various commercial IVUS systems without the need for any special software such as VH-IVUS. The RF-image combined approach has potential clinical applicability, since it complements the abovementioned problems. However, only a few studies [27, 33] have been carried out to date examining this, and the increasing computational time remains an unsolved problem. Therefore, the purpose of this study was to automatically characterize DC tissue in the gray scale IVUS images based on the image textural features.

## Methods

### Patients

We retrospectively analyzed patients with acute coronary syndrome who have undergone IVUS examination along with X-ray angiography at our hospital during a 5-year period (October 192,009 to September 302,014). One experienced cardiologist with over 20 years of experience in cardiovascular diagnosis performed all procedures and reviewed the results. The inclusion criteria for the study included (1) availability of both the IVUS and X-ray angiography; (2) diagnosed with acute coronary syndrome; and (3) enough image quality of IVUS images. The exclusion criteria were as follows: 1) patients with a stent implantation; 2) patients with a bypass graft, and 3) poor image quality. After screening, a total of 316 Gy-scale IVUS and their corresponding VH-IVUS images were obtained from 26 patients with 26 lesions. All lesions were located in right coronary artery. This study was approved by the local ethics committee. Due to the retrospective nature of the study, written informed consent was waived.

### Image data acquisition

**Fig. 1:**
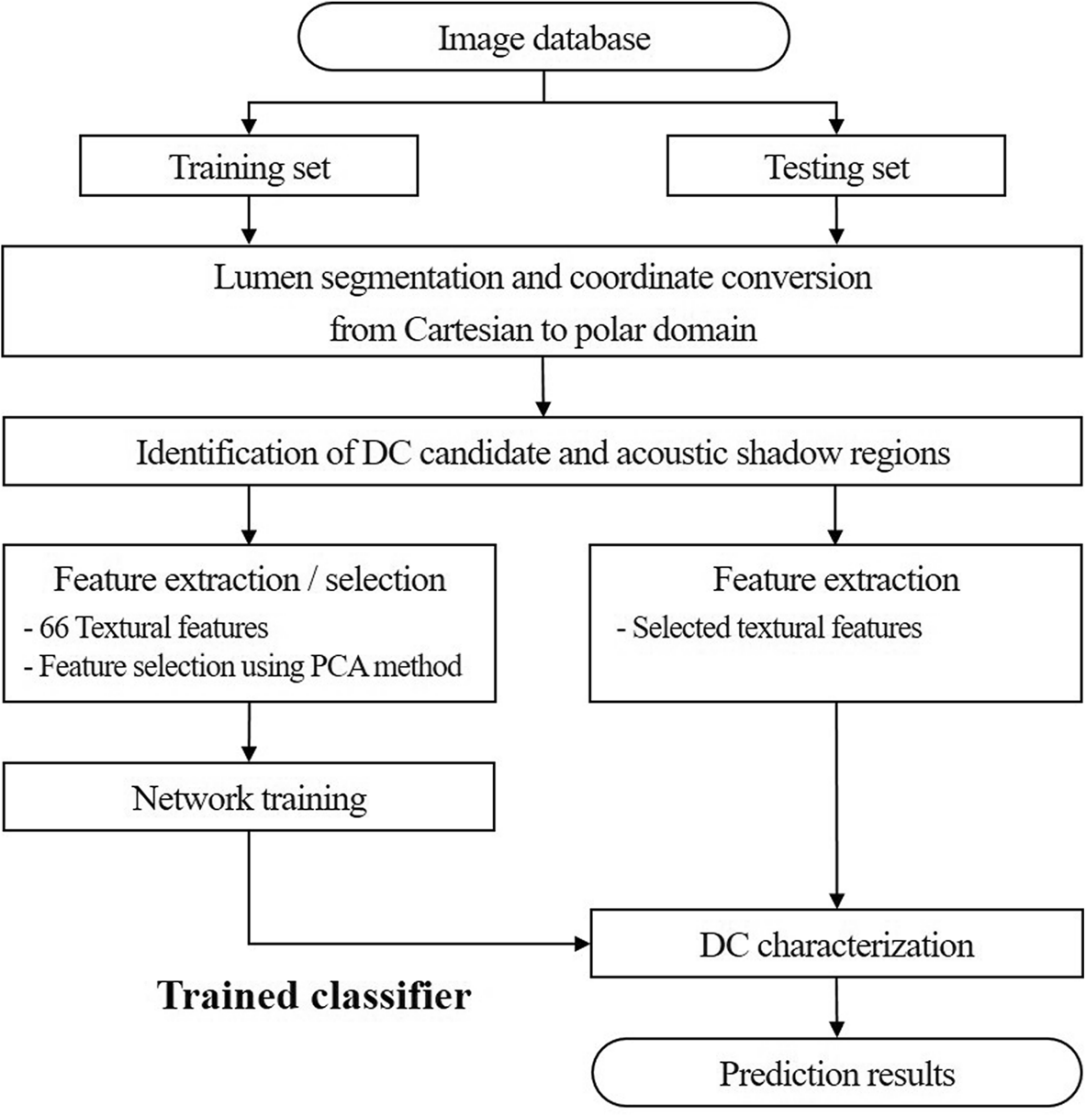
Overall workflow of the proposed method for DC characterization (DC: dense calcium, and PCA: principal component analysis). The proposed method has two slightly different pipelines for training and testing data sets. Feature selection was only performed on the training set. When testing the network, only the selected features were extracted

Prior to image acquisition, the plaque regions from intima to media-adventitial borders were automatically determined using our previously proposed method [34] and manually corrected by the clinician. VH analysis was performed only on these plaque regions and the output images were stored in the BMP format. Figure 1 shows the overall tissue characterization procedure of the proposed method.

IVUS imaging was performed using an imaging system incorporating a commercially available 20 MHz Eagle Eye catheter (Volcano Therapeutics Inc., Rancho Cordova, CA, USA). The catheter was advanced over a conventional guidewire until reaching the lesion of interest, and the catheter position was validated using X-ray angiography. The pullback was performed with a pullback speed of 0.5 mm/s, acquiring 30 frames/s during image acquisition. IVUS images were recorded along with a simultaneous ECG at 400 × 400 pixels in 8-bit grayscale.

The attained VH-IVUS images typically exhibited different proportions of the plaque components in the order of FT > FFT > NC > DC. In order to avoid any possibility of biased training of the classification model, their proportions were adjusted to a 1:1 ratio. Consequently, the training data set contained approximately 3,000,000 labeled pixels and these were divided into non-DC and DC groups in equal proportion. Non-DC group comprises FT (500,000 pixels), FFT (500,000 pixels), and NC (500,000 pixels), while the DC group only includes NC tissue (1,500,000 pixels). Each pixel in the input gray-scale IVUS images was classified into one of two groups on the basis of the related ground truth (VH-IVUS).

### Segmentation of DC candidate and acoustic shadow regions

**Fig. 2:**
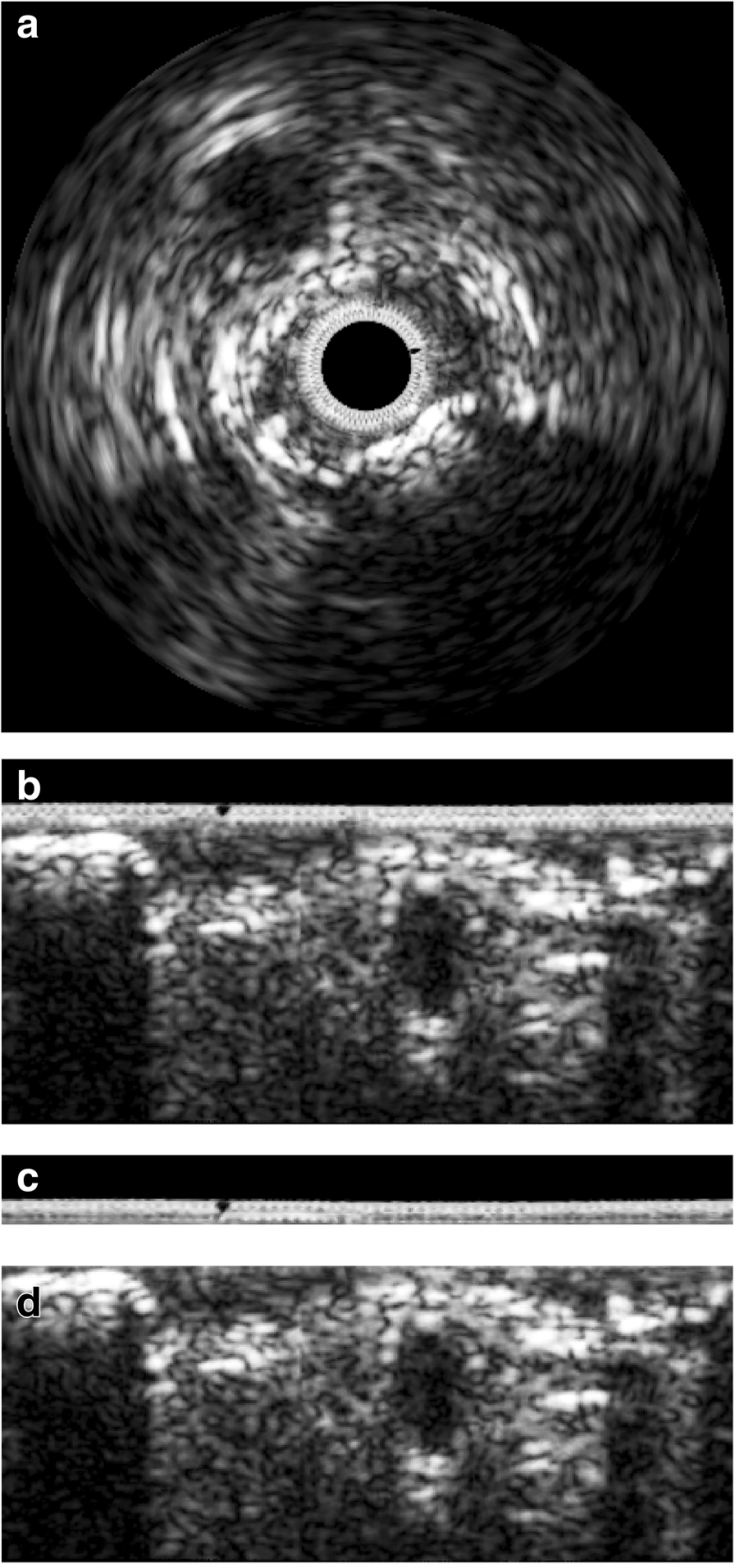
**a** The original IVUS image in Cartesian coordinate, (**b**) converted IVUS image in polar coordinate, (**c**) constant dead zone, (**d**) subtracted image

The DC candidate and corresponding acoustic shadow regions were automatically detected by the dual-threshold-based segmentation method [32] prior to tissue characterization. The original IVUS image in Cartesian coordinates is relatively difficult to handle due to the circular trait of vessels (Fig. 2a). In order to simplify the segmentation procedure, the IVUS image was converted into polar coordinates (Fig. 2b). The catheter typically induces an unnecessary dead zone in the center of the IVUS image, equivalently at the top rows of the polar domain with imaging artifacts. Therefore, the constant dead zone was acquired by calculating the minimum image over 316 IVUS sequences (Fig. 2c), and this was subtracted from every frame in order to avoid interference of the catheter (Fig. 2d).

**Fig. 3:**
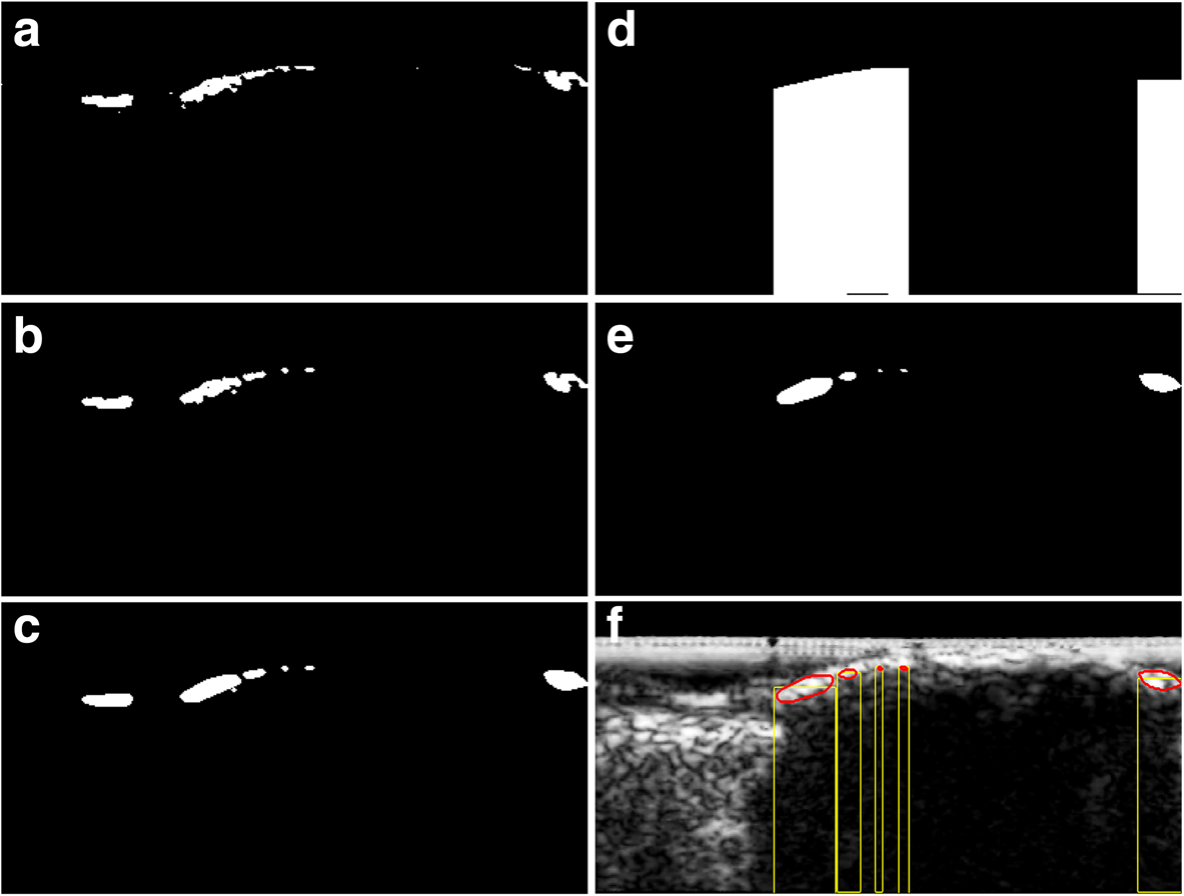
**a** The initial DC candidates, (**b**) morphological operations, **c** convex hull, (**d**) vertical shadow mask, (**e**) final DC candidate, and (**f**) finally obtained DC candidate with corresponding acoustic shadow

The initial DC candidates were obtained by extracting regions having higher pixel intensity than *TH*_*high*_ in plaque regions (Fig. 3a):

1 $$ {R}_{IDC}\left(i,j\right)=\sum \left\{\left({I}_{IDC}\left(i,j\right)>{TH}_{High}\right)\right\}\in PR $$

where *R*_*IDC*_ and *I*_*IDC*_*(i,j)* indicate the initial DC candidate region and the intensity of the pixel *(i, j)*, respectively. High threshold (*TH*_*High*_) detects the initial DC candidate, and *PR* denotes the plaque region. In order to remove noises in the output image, morphological operations, comprising erosion and dilation, were subsequently used (Fig. 3b). In terms of mathematical morphology, a structuring element was a disk at a size of 1 × 1 pixel. As depicted in Fig. 3b, there are numerous DC candidates which are distributed very closely and may have similar or identical acoustic shadows. Therefore, the convex hull of these regions was computed using the plane sweep method [35] in order to minimize the computational time when the shortest distance of adjacent candidate was less than 10 pixels (Fig. 3c).

The resulting regions were assigned to the final DC candidate or non-calcified classes according to the presence or absence of shadow. The average intensity of the vertical shadow mask having the same width as the output candidate was calculated (Fig. 3d) and the pixels that satisfied *I*_*AS*_ *< T*_*Low*_ were finally considered as the final DC candidates (Fig. 3e). Consequently, only the region that satisfied the following dual threshold condition was accepted as the final DC candidate.

2 $$ {R}_{DC}\left(i,j\right)=\sum \left\{\left({I}_{IDC}\left(i,j\right)>{TH}_{High}\right)\  and\ \left({I}_{AS}\left(i,j\right)<{TH}_{Low}\right)\right\}\in PR $$

where *R*_*DC*_ and *I*_*AS*_ are the final DC candidate and intensities of the acoustic shadow, respectively. Low threshold (*TH*_*Low*_) assesses the existence of shadow. The dual-threshold values of *TH*_*High*_ and *TH*_*Low*_ were empirically selected at 160 and 50, respectively, by computing the mean intensity of DC and the shadow regions in all of the IVUS images. Figure 3f depicts the finally obtained DC candidate with its corresponding shadow region.

### Feature extraction

In order to accurately detect the DC plaque, nine image-based feature groups comprising 66 different features were automatically extracted from a total of 3,000,000 pixels in the plaque regions by shifting a moving window from the top left to the bottom right of each image. Each feature extraction method was selected for its successful report of tissue characterization in two-dimensional (2D) gray-scale images. A set of all features was extracted for each pixel in the DC candidate using a 5 × 5 moving window.

#### First order statistics (FOS)

Statistical features derived from FOS are significant indicators of spatial relationship. FOS analyzes the image characteristics based on the gray-level distribution histogram. Five features, namely mean, skewness, kurtosis, variance, and standard deviation were extracted from a 5 × 5 window mask. Detailed descriptions and equations are referred to in [36, 37].

#### Intensity

**Fig. 4:**
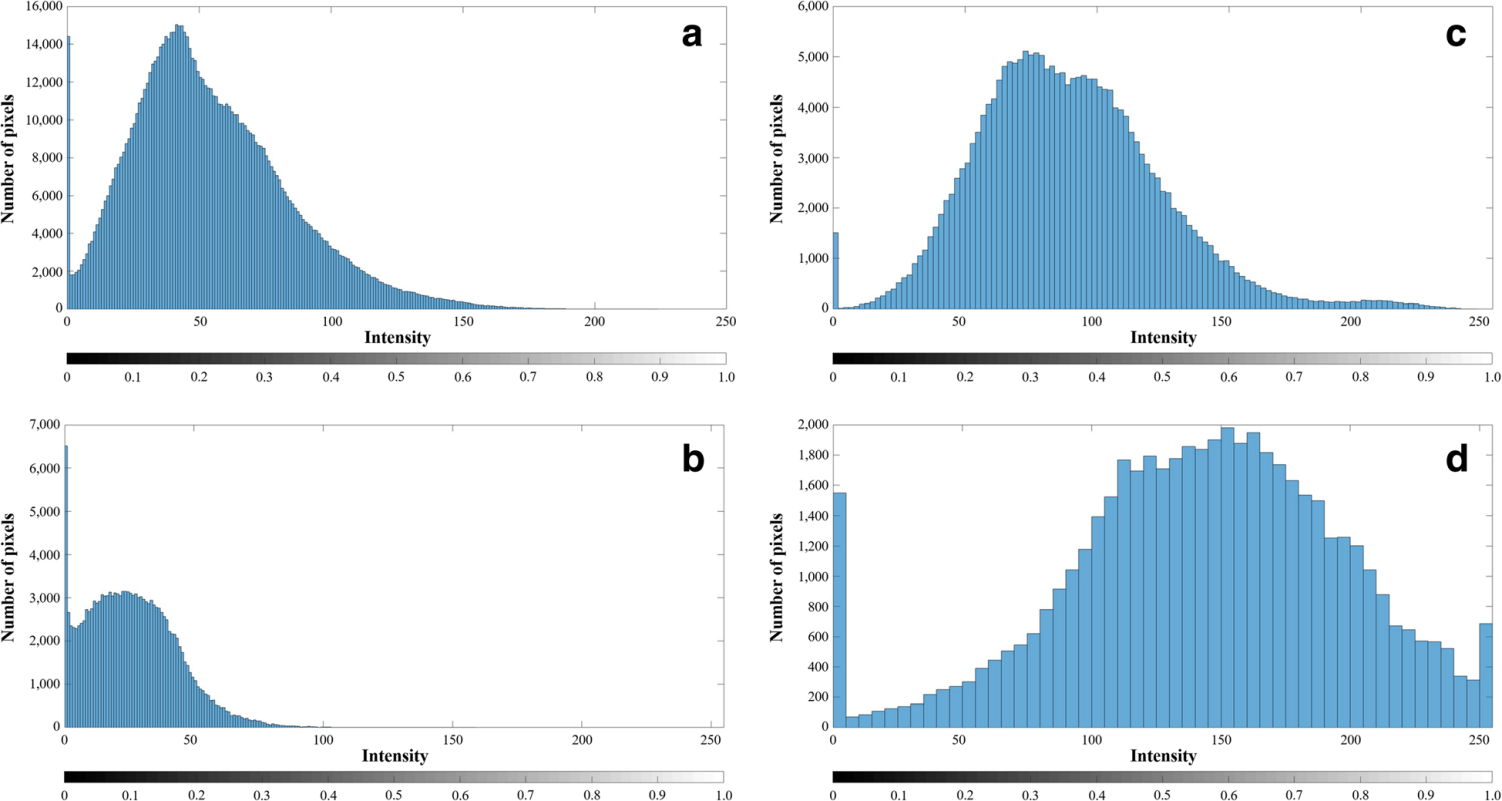
Intensity distributions of four plaque components. **a** is fibrous tissue (FT), (**b**) is fibrofatty tissue, (**c**) is necrotic core, and (**d**) is dense calcium

Intensity is the gray-scale value of each pixel obtained by the amplitude of the reflected ultrasound RF signal from plaque components [38]. As reported in [30], the plaque components typically have a different intensity distribution whereby NC and DC shows higher echo-intensities than FT and FFT (Fig. 4). Additionally, DC contains the highest intensity components due to its echogenic characteristic.

#### Geometrical distance features (GDF)

The GDF indicates a Euclidean distance of each DC candidate from a specific location and can be divided into three sub-features (GDF_1_, GDF_2_, and GDF_3_). First, the GDF_1_ describes the perpendicular distance of a pixel that belongs to the DC candidates from the center of the catheter. The second geometrical feature, GDF_2_, is the relative position of the DC candidate pixel from the media-adventitial borders, while the GDF_3_ indicates the relative position from the lumen and media-adventitial borders [1].

#### Fractal dimension (FD)

The FD is a measure of complexity that quantifies the fractal patterns of its intensity surface as the ratio of the change in detail to the change in scale. Despite not being self-similar over all of the scales, the reflected RF signals from plaque components typically generate some level of self-similarity within some range [39]. Therefore, the fractal dimensions of plaque components can be a significant indicator for the differentiation of DC tissues. In this paper, the FD of tissue component patterns was computed using the box counting method [40, 41].

#### Gray Level Co-Occurrence Matrix (GLCM)

GLCM estimates the co-occurrence values of gray-level pairs from angular nearest-neighbor spatial-dependence matrices [1, 42]. For a fixed window size, the co-occurrence matrix (*CM*) for an *h x w* plaque region (*PR*) can be defined as follows:

6 $$ {CM}_{\Delta  x,\Delta  y}\left(i,j\right)=\sum \limits_{x=1}^h\sum \limits_{y=1}^w\frac{1, if\  PR\left(x,y\right)=i\  and\  PR\left(x+\Delta  x,y+\Delta  \mathrm{y}\right)=j}{0, otherwise} $$

where *i* and *j* denote the pixel values and *x* and *y* indicate the spatial positions in *PR*. The offsets *(∆ x, ∆ y)* determine the spatial relations of the calculated matrix. In our experiments, the distance was set to *D = 1* and the orientation angle was designated as one of four values (0°, 45°, 90°, or 135°). The features obtained by the different orientations were then averaged in order to guarantee rotational invariance. A total of 19 features comprising autocorrelation, contrast, correlation, cluster prominence, cluster shade, dissimilarity, energy, entropy, homogeneity, maximum probability, sum of squares, sum average, sum variance, sum entropy, difference variance, difference entropy, information measure of correlation, maximal correlation coefficient, and inverse difference normalized, were computed from the GLCM matrix [42].

#### Gray level run length matrix (GLRLM)

GLRLM features provide the textural patterns in the plaque regions by analyzing the relation between gray-level values appearing along pixel sequences. For a given plaque region, each feature for a certain pixel was extracted based on the element of GLRLM. From the vertical and horizontal directions, a total of 11 GLRLM features comprising short run emphasis, long run emphasis, gray-level non-uniformity, run-length non-uniformity, run percentage, low gray-level run emphasis, high gray-level run emphasis, short run low gray-level emphasis, short run high gray-level emphasis, long run low gray-level emphasis, and long run high gray-level emphasis were extracted and averaged in each pixel. Detailed descriptions and equations are referred to in [21, 22].

#### Neighborhood gray-tone difference matrix (NGTDM)

NGTDM features reflect the spatial changes in intensity between the gray level of a certain pixel and the average of its neighborhood gray levels. This matrix converts the original 2D images into a column matrix by averaging the differences between the gray level of the center pixel and that of the surrounding neighbors. In this study, five features were obtained from a column matrix: coarseness, contrast, busyness, complexity, and texture strength [43]. The features are referred to in detail in [24, 44, 45].

#### Law’s texture energy (LTE)

LTE is known involves energy-based texture feature and computes the amount of variation within a 5 × 5 convolution kernel. LTE features can be obtained through four simple vectors of L5 (Level), E5 (Edge), S5 (Spot), and R5 (Ripple) [46]. Four coefficients were multiplied in order to form 2D kernels (L5E5/E5L5, L5S5/S5 L5, L5R5/R5L5, E5S5/S5E5, E5R5/R5E5, R5S5/S5R5, S5S5, E5E5, and R5R5), and these kernels were convolved with the input images. Each kernel formed from orthogonal matrices, such as L5S5 and S5 L5, were subsequently averaged in order to reflect rotational invariance [24]. In this paper, the sum of the squared value and the sum of the absolute value [46] of the image were computed for all convolution kernels. Consequently, a total of 18 features were extracted from LTE maps.

#### Local binary pattern (LBP)

LBP is a non-parametric measure used to efficiently characterize the local structures by allocating a binary number to the circularly symmetric neighborhoods of the center pixel [47]. LBP compares the intensity of the central pixel with the intensities of the surrounding pixels. The neighboring pixels are then thresholded with a central pixel value and the results are regarded as a binary number. In our experiments, the three LBP features of Rotation Invariant, Uniform Rotation Invariant, and Local Variance were extracted. The features are referred to in detail in [3, 22].

### Feature selection

Not all of the image-based features are helpful for tissue characterization, and some may negatively influence the classification results [48, 49]. In order to reduce the dimensionalities and select optimum feature sets, principal component analysis (PCA) was implemented followed by a varimax rotation. PCA is a statistical method that converts a set of the original features into a set of values of linearly uncorrelated variables called principal components. The selected feature sets reduce the computational load while not actually degrading the classification performance, owing to the linear combinations. The primary principal component represents the direction in which the features vary most, and the second component describes the next largest amount of variance which is not related to the primary one. Hence, it is possible to condense most of the useful information into the first few components. In this study, the first principal component was used to select optimum features, and the classification accuracies were then quantitatively evaluated.

### DC characterization using deep belief network (DBN)

A deep neural network initialized by a DBN was implemented as a classification model for DC characterization. The basic concept of DBN is to use a layer-wise unsupervised learning method in order to pre-train the initial weight values of the network [50]. The DBN model is a generative graphical model that can learn to probabilistically reconstruct the inputs; it is composed of multiple layers of latent variables with connections between the layers but not between units within each layer [51].

The training of the DBN model was carried out in two steps: pre-training and supervised fine-tuning. First, each pair of layers in the network was pre-trained using a restricted Boltzmann machine (RBM). RBM is a generative stochastic and two-layered neural network where only inter-layer connections are allowed. This model is able to learn a probability distribution over its inputs [52]. The main advantage of the RBM is that the hidden units are conditionally independent, because there are no connections within any layers. At the beginning of learning, the weights were initialized to values sampled from a Gaussian distribution with mean 0 and a standard deviation of about 0.01. The visible and hidden biases were initialized to 0 and standard deviation of 0.01. The learning was performed with a batch size of 100 samples and was completed over 10 epochs. Detailed descriptions of RBM are referred to in [53, 54].

Following the unsupervised training of RBM, the classifier was refined using the standard supervised feed-forward neural network (FFNN). The output weights of the DBN were transferred to a multi-layer FFNN with the same number of input, hidden, and output neurons, and the values were fine-tuned using the back-propagation method. The rectified linear and softmax units were used as activation functions of the hidden and output layers, respectively. The dropout fraction was set to 0.5 during the training process. Using back-propagation that was initially learned as a generative stochastic model led to much better performance than using back-propagation with randomly initiated weights in the traditional neural network. The random forest (RF), Adaboost, support vector machine (SVM), FFNN, and radial basis function neural network (RBFNN) were used to evaluate the performance superiority of the proposed method.

### Performance evaluation

The proposed method was evaluated using the 10-fold cross validation in order to avoid the influences which were attributable to similar tissue characteristics of the same pullback images. The selected features were divided into ten independent sub-samples followed by each pullback and one of these groups was used to test other sub-sample groups while the others were used as training sets. This process was repeated ten times so that all pullbacks are used as both training and test sets. For training and testing the network, VH-IVUS images were used as the ground truth data.

For each iteration, the classification performance was quantitatively evaluated in terms of specificity, sensitivity, positive predictive value (PPV), negative predictive value (NPV), and accuracy. The classification results were also validated through receiver operating characteristic (ROC) analysis. The ROC curve demonstrates the statistical correlations between the sensitivity and the specificity based on the varying thresholds. The area under the ROC curve (AUC) was computed for each iteration. All image processing including feature extraction, selection, training and testing the neural network was performed using MATLAB software package (R2015b, MathWorks Inc., Natick, MA, USA) on a NVIDIA GeForce GTX 1080 Ti GPU with 64 GB of RAM.

### Statistical analysis

We performed a student’s t-test to evaluate the statistical significance between ground truth data and predicted result. Data were described as the means±standard deviations (SD). *P* values less than 0.05 were considered statistically significant. All statistical analyses were performed using the SPSS Statistics 17.0 (SPSS Inc., Chicago, IL, USA) software.

## Results

### Feature selection using PCA

A total of 66 textural sub-features were extracted from the eight feature sets of FOS, intensity, GDF, GLCM, GLRLM, NGTDM, LTE and LBP. These were then optimized using the PCA method and only 33 features were obtained, as listed in Table 1. Mean and variance were selected from FOS group, and 14 features remained from 19 GLCM features. For GLRLM and LTE, only sub-features 4 and 11 were survived, respectively. Additionally, the intensity and fractal dimension, which plays an important role in DC characterization, were determined to be the optimum feature set. On the other hand, the textural features of GDF, NGTDM, and LBP were all excluded. The selected feature set was utilized as input for DBN, and the classifier categorized each pixel into either calcified or non-calcified tissues.

**Table 1:** Selected feature subsets from the original features using PCA method. Only 33 features were selected as the best feature set for DC characterization and these were used as the input for classification model (FOS: first order statistics, FD: fractal dimension, GLCM: gray level co-occurrence matrix, GLRLM: gray level run length matrix, and LTE: Law’s texture energy)

No.	Feature Group	Feature Subsets
1	FOS	Mean
2		Variance
3	Intensity	Intensity
4	FD	Fractal Dimension
5	GLCM	Difference Variance
6		Contrast
7		Sum Variance
8		Autocorrelation
9		Cluster Prominence
10		Sum of Squares
11		Sum Average
12		Entropy
13		Energy
14		Homogeneity
15		Maximum Probability
16		Sum Entropy
17		Dissimilarity
18		Difference Entropy
19	GLRLM	SRE
20		LRE
21		GLN
22		HGRE
23	LTE	(SSV) R5S5/S5R5
24		(SSV) E5E5
25		(SSV) E5S5/S5E5
26		(SSV) E5R5/R5E5
27		(SSV) S5S5
28		(SSV) R5R5
29		(SAV) R5S5/S5R5
30		(SAV) S5S5
31		(SAV) R5R5
32		(SAV) E5R5/R5E5
33		(SAV) L5S5/S5 L5

### DC characterization results

Although there were some improvements in NPV and sensitivity, the results showed no significant differences in any evaluation metrics with or without the PCA method (*p* > 0.05). This result indicates that the dimensionality of the feature set was efficiently decreased without remarkable performance degradation. Table 2 demonstrates the DC characterization results of the proposed method in terms of PPV, NPV, sensitivity, specificity, accuracy, and AUC, without and with PCA method.

**Table 2:** DC characterization results in terms of PPV, NPV, sensitivity, specificity, accuracy and AUC without and with PCA method. *P*-value was obtained using student’s t-test between ground truth data and predicted results (PPV: positive predictive value, NPV: negative predictive value, and AUC: area under the ROC curve)

Mask Size	PPV (%)	NPV (%)	Sensitivity (%)	Specificity (%)	Accuracy (%)	AUC
3 × 3	86.7 ± 0.1	82.5 ± 0.1	81.6 ± 0.1	87.3 ± 0.1	84.5 ± 0.1	0.846 ± 0.002
7 × 7	85.4 ± 0.1	91.8 ± 0.1	92.5 ± 0.1	84.1 ± 0.1	88.3 ± 0.1	0.886 ± 0.001
9 × 9	89.1 ± 0.1	84.0 ± 0.1	82.9 ± 0.1	89.8 ± 0.1	86.3 ± 0.1	0.865 ± 0.001
11 × 11	87.1 ± 0.1	87.4 ± 0.1	87.6 ± 0.1	87.0 ± 0.1	87.3 ± 0.1	0.873 ± 0.001
5 × 5	86.0 ± 0.1^†^	91.2 ± 0.1^*†ψ^	92.8 ± 0.1^*†ψ^	85.1 ± 0.1	88.4 ± 0.1^*†^	0.886 ± 0.001^*†^

**Fig. 5:**
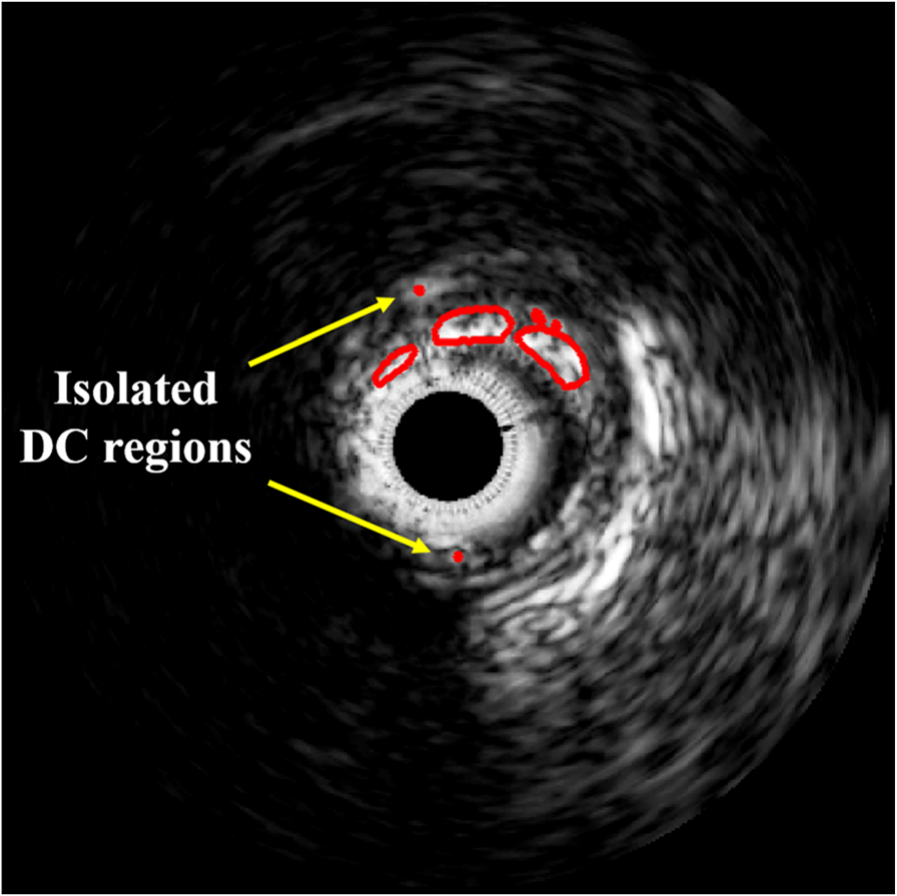
The isolated small DC regions which showed the poorest classification results

**Fig. 6:**
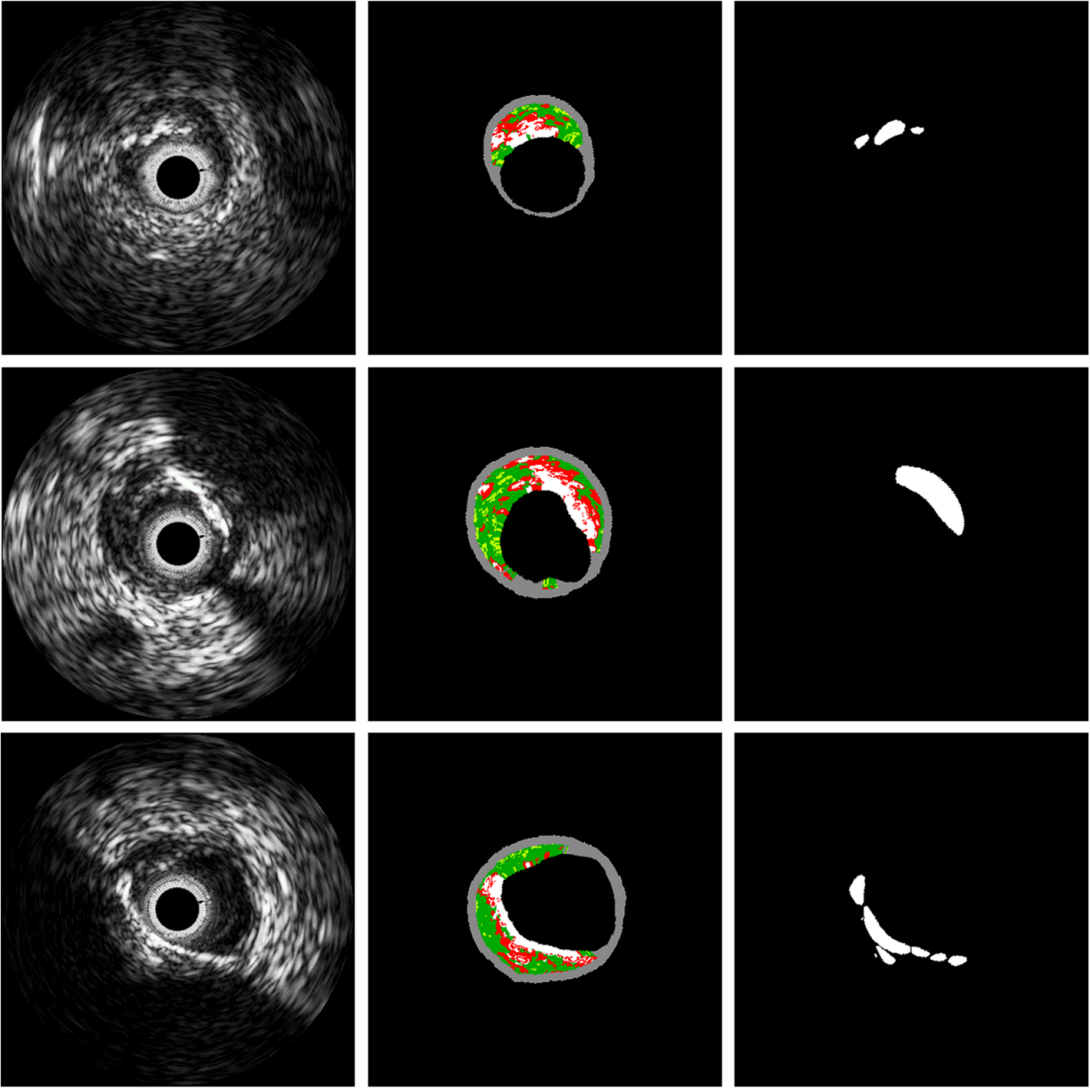
Classification results mapped to (x, y) view. Panels show: (left) original image, (middle) corresponding VH-IVUS, and (right) predicted result. Colors are green (fibrous tissue), light green (fibro-fatty tissue), red (necrotic core), and white (dense calcium)

For varying window sizes, the condition of 3 × 3 mask size not only had the poorest sensitivity and accuracy, but also showed isolated regions as shown in Fig. 5. On the other hand, the 5 × 5 and 7 × 7 window sizes presented similar classification results, and the sensitivity drastically decreased by nearly 10% as the size increased afterwards (Table 3). Based on these results, it was found that the window size can largely influence the classification results and that the 5 × 5 window size was the best for plaque characterization. The DC characterization results are shown in Fig. 6. The predicted classification maps agreed favorably with the VH-IVUS counterparts.

**Table 3:** Classification results according to the different window sizes of 3 × 3, 5 × 5, 7 × 7, 9 × 9, and 11 × 11. The condition of 3 × 3 mask size had the worst results, while the condition of 5 × 5 provided the best results. This result indicates that the mask size can largely influence the classification performance. Statistically significant differences (p < 0.05) compared with ‘3 × 3’, ‘7 × 7’, ‘9 × 9’, and ‘11 × 11’ are indicated by ‘*’, ‘γ’, ‘†’, and ‘ψ’, respectively, as determined from the student’s t-test

Mask Size	PPV (%)	NPV (%)	Sensitivity (%)	Specificity (%)	Accuracy (%)	AUC
3 × 3	86.7 ± 0.1	82.5 ± 0.1	81.6 ± 0.1	87.3 ± 0.1	84.5 ± 0.1	0.846 ± 0.002
7 × 7	85.4 ± 0.1	91.8 ± 0.1	92.5 ± 0.1	84.1 ± 0.1	88.3 ± 0.1	0.886 ± 0.001
9 × 9	89.1 ± 0.1	84.0 ± 0.1	82.9 ± 0.1	89.8 ± 0.1	86.3 ± 0.1	0.865 ± 0.001
11 × 11	87.1 ± 0.1	87.4 ± 0.1	87.6 ± 0.1	87.0 ± 0.1	87.3 ± 0.1	0.873 ± 0.001
5 × 5	86.0 ± 0.1^†^	91.2 ± 0.1^*†ψ^	92.8 ± 0.1^*†ψ^	85.1 ± 0.1	88.4 ± 0.1^*†^	0.886 ± 0.001^*†^

### Comparison with other existing classification models

In order to evaluate the performance superiority of the DBN classifier, the classification results of RF, Adaboost, SVM, FFNN, and RBFNN were quantitatively compared in terms of all evaluation metrics. All classification models revealed a very small accuracy difference within 2% compared to the DBN model (*p* > 0.05). Although the conventional machine learning approaches, such as RF, Adaboost, and SVM, slightly improved the specificity and accuracy, the sensitivity was decreased by over 4% (*p* < 0.05). FFNN had lower values of specificity and accuracy than the proposed method, whereas the superior DC classification results were obtained as the sensitivity was improved by about 2.4%. The RBFNN model revealed the lowest sensitivity for all employed classifiers. According to these results, it was confirmed that the optimized feature set of the proposed method was properly determined using the PCA method. Table 4 shows the DC characterization results for different classification methods.

**Table 4:** DC characterization results for different classification methods. All methods had a very small accuracy difference within 2% compared to the proposed method. Statistically significant differences (p < 0.05) compared with ‘RF’, ‘Adaboost’, ‘SVM’, ‘FFNN’, and ‘RBFNN’ are indicated by ‘*’, ‘γ’, ‘†’, ‘ψ’, and ‘δ’, respectively, as determined from the student’s t-test. Data are shown as mean ± standard deviation

Methods	PPV (%)	NPV (%)	Sensitivity (%)	Specificity (%)	Accuracy (%)	AUC
RF	87.1 ± 0.1	87.7 ± 0.1	87.1 ± 0.1	87.8 ± 0.1	87.4 ± 0.1	0.874 ± 0.003
Adaboost	87.1 ± 0.1	86.3 ± 0.1	87.3 ± 0.2	86.1 ± 0.1	86.7 ± 0.1	0.867 ± 0.002
SVM	87.7 ± 0.1	89.8 ± 0.1	88.0 ± 0.0	89.6 ± 0.0	88.8 ± 0.1	0.888 ± 0.001
FFNN	83.6 ± 0.2	93.3 ± 0.1	94.2 ± 0.3	81.4 ± 0.1	87.8 ± 0.2	0.885 ± 0.001
RBFNN	85.9 ± 0.1	89.8 ± 0.1	85.1 ± 0.1	90.3 ± 0.1	87.7 ± 0.1	0.878 ± 0.001
Proposed Method	86.0 ± 0.1^ψ^	91.2 ± 0.1^*γ^	91.8 ± 0.1^*γ†δ^	85.1 ± 0.1^†ψδ^	88.4 ± 0.1	0.886 ± 0.001

## Discussion

None of GDF, NGTDM, and LBP features were selected during feature selection using the PCA method. GDF is an indicator that presents the relative position of DC tissues in the plaque region. In their previous study [1], Athanasiou et al. reported that geometrical features could improve the classification accuracy at 2.35%. Despite this improvement, one of the possible reasons for exclusion is an inaccurate MA border caused by acoustic shadow. It is considered that GDF_2_ and GDF_3_ showed low significance levels, because these features were influenced by the border locations. Typically, coronary arteries are highly prone to rupture when more DC tissues are distributed close to the intima border. Some studies have reported that the location of each tissue from the vessel lumen is a useful index for discriminating DC tissues [9, 55]. Although it was validated with optical coherence tomogram (OCT), which can measure the thickness of the fibrous cap, it is quite possible to apply in IVUS images as well. Therefore, further studies are needed to determine the applicability. NGTDM and LBP were considered as excellent indicators for expressing textural patterns of the image [2, 3, 21, 43–45]. However, these descriptors not only present only a small change in the output when the change is small in the input image, but are also not robust for noisy images [56]. This is due to the thresholding scheme of the operator. Unlike OCT images, IVUS images has numerous speckle noises and small changes of values because of pixel-to-pixel relations. These inherent characteristics resulted in low significances.

Although the PCA method slightly improved classification performance, there was no drastic improvement for DC characterization. However, the main reason for using feature selection is to reduce the feature dimensionality rather than improve the classification accuracy. The proposed method not only reduced the feature dimensionality over 50%, but also slightly improved the sensitivity level simultaneously. The selected features revealed similar classification results for 10-fold cross validation, and we found a significant reliability for these textural features. With regard to the window size, the size of 3 × 3 showed the lowest sensitivity, because this mask includes a relatively small number of image pixels. The excessive large window conditions over 9 × 9 size also had poor classification results and this was noticeably shown in the regions where the small DC tissues are isolated. Small windows are able to preserve detailed information and avoid the influences of adjacent pixels (tissues) but may include limited data for determining tissue type. On the other hand, large windows tend to result in misleading texture information, particularly for border regions. The window sizes of 5 × 5 and 7 × 7 showed similar levels of classification. However, the condition of 5 × 5 was selected as the optimum mask condition, since this condition is advantageous over other conditions in terms of computational load.

This study implemented the DBN model for discriminating DC tissues and compared the classification results with various classifiers including RF, Adaboost, SVM, FFNN, and RBFNN, under the same feature sets. Consequently, the conventional models of RF, Adaboost, and SVM revealed over 87% of sensitivity, while the FFNN representing typical aspects of neural network showed the highest sensitivity (94.22%). These results indicate that the feature selection was carried out quite successfully. Nevertheless, we implemented the DBN model in order to cope with larger data sets and preserve the fastest processing speed. The DBN model has four main advantages: (1) it is fine-tunable, (2) it is generatively pre-trainable, (3) includes many hidden layers, and (4) allows for dimensionality reduction for input features [57]. For 3 to 5 million pixels, most existing classifiers may produce reliable results. However, the traditional models are difficult to be trained with excessively large datasets due to the vanishing gradient, overfitting, and excessive computational load. Among the existing methods, it is worth analyzing the applicability of the convolutional neural network (CNN). The CNN model classifies on the basis of pre-determined masks without any feature extraction process. The CNN model was not considered in this study, because it is not able to reflect the inherent tissue properties in medical images. However, if the clinical, textural, and CNN properties are combined and utilized as input features, it may improve the classification performance drastically.

In our previous study in [32], the DC and corresponding acoustic shadow regions were automatically detected from the gray-scale IVUS images using only the dual-threshold-based segmentation without any specific classification model. Despite the significant similarities, the output regions contained a substantial part of NC tissue (28.4%), since the NC frequently accompanies acoustic shadows. This result indicates that the sole intensity information cannot be a good indicator for the DC classification, although the DC tissues have visually distinct intensity characteristics. In order to solve this problem, this study designated these regions as the DC candidate and classified each pixel to calcified or non-calcified tissues by analyzing the textural feature patterns. As a consequence, the proposed method improved the sensitivity up to 92.8% and all of the remaining (7.2%) belonged to the NC group. The reason for the misleading of NC tissues comes from the fact that some parts of NC tissues have overlapped intensity ranges with DC tissues (Fig. 4). It is necessary to perform the RF-based analyses, such as wavelet analysis, rather than the image-based analysis in order to improve the classification accuracy.

With our fully-automated method, one can run the entire pullback or manually set the start and end frames, and then review results. The proposed method also has high clinical applicability in terms of computational time. On our computer system with non-optimized code, the total amount of time necessary to perform the whole process from the lumen segmentation to DC classification was around 0.7 s (0.1 s for lumen segmentation, 0.1 s for determining DC candidates, 0.5 s for feature extraction, and 0.02 s for classification). Considering that each pullback usually includes 100–200 frames, the proposed method can complete the entire DC characterization within 70–140 s.

This study has three main limitations: First, the proposed method was still not able to perfectly discriminate DC tissues from NC tissues, despite the relatively high sensitivity. Second, the VH-IVUS was regarded as the ground truth. As previously reported in [5], VH-IVUS shows a reliable classification accuracy of over 93% for cardiovascular tissues. However, it may potentially cause misleading results, since VH-IVUS still has minor errors. Third, it is difficult to quantify the amount of DC tissues due to the high reflectance of ultrasound signal, giving indeterminate pixels in an image.

## Conclusions

This study proposed a fully automated classification method for DC tissues in the gray-scale IVUS images. We determined the best feature set and window size for DC characterization. The proposed method had significantly high levels of sensitivity, accuracy, and AUC. DBN model offered better characterization performance than other traditional models. Experimental results confirmed that the proposed method has high clinical applicability for IVUS-based cardiovascular diagnosis. Particularly, our method only required about 0.7 s per image for DC characterization, indicating that it only takes few minutes for the entire pullback. These advantages would enable the cardiologists to expedite the interventional decision making. Future research should include histopathological experiments to automatically classify FT, FFT, NC, and DC in the plaque regions. Moreover, the feasibility of combined features of IVUS and OCT images for tissue characterization should be assessed.

## Data Availability

The datasets used in this study are available from the corresponding author on reasonable request.

## Abbreviations

2D: two dimensional
AUC: Area under the ROC curve
CNN: Convolutional neural network
DBN: Deep belief network
DC: Dense calcium
ECG: Electrocardiogram
FD: Fractal dimension
FFNN: Feed-forward neural network
FFT: Fibro-fatty tissue
FOS: First order statistics
FT: Fibrous tissue
GDF: Geometrical distance features
GLCM: Gray level co-occurrence matrix
GLRLM: Gray level run length matrix
IVUS: Intravascular ultrasound
LBP: Local binary pattern
LTE: Law’s texture energy
NC: Necrotic core
NGTDM: Neighborhood gray-tone difference matrix
NPV: Negative predictive value
OCT: Optical coherence tomogram
PCA: Principal component analysis
PPV: Positive predictive value
RBFNN: Radial basis function neural network
RBM: Restricted Boltzmann machine
RF: Radio frequency
RF: Random forest
ROC: Receiver operating characteristic
SD: Standard deviation
SVM: Support vector machine
VH: Virtual histology
